# A Light-Powered Self-Circling Slider on an Elliptical Track with a Liquid Crystal Elastomer Fiber

**DOI:** 10.3390/polym16162375

**Published:** 2024-08-22

**Authors:** Lu Wei, Yanan Chen, Junjie Hu, Xueao Hu, Jiale Wang, Kai Li

**Affiliations:** School of Civil Engineering, Anhui Jianzhu University, Hefei 230601, China; weilu@ahjzu.edu.cn (L.W.); cyn@stu.ahjzu.edu.cn (Y.C.); jjhu@stu.ahjzu.edu.cn (J.H.); hxa@stu.ahjzu.edu.cn (X.H.); wjl1230504@163.com (J.W.)

**Keywords:** self-circling, liquid crystal elastomer, light powered, slider, elliptical track

## Abstract

In this paper, we propose an innovative light-powered LCE-slider system that enables continuous self-circling on an elliptical track and is comprised of a light-powered LCE string, slider, and rigid elliptical track. By formulating and solving dimensionless dynamic equations, we explain static and self-circling states, emphasizing self-circling dynamics and energy balance. Quantitative analysis reveals that the self-circling frequency of LCE-slider systems is independent of the initial tangential velocity but sensitive to light intensity, contraction coefficients, elastic coefficients, the elliptical axis ratio, and damping coefficients. Notably, elliptical motion outperforms circular motion in angular velocity and frequency, indicating greater efficiency. Reliable self-circling under constant light suggests applications in periodic motion fields, especially celestial mechanics. Additionally, the system’s remarkable adaptability to a wide range of curved trajectories exemplifies its flexibility and versatility, while its energy absorption and conversion capabilities position it as a highly potential candidate for applications in robotics, construction, and transportation.

## 1. Introduction

Self-sustained oscillation [1,2] refers to the spontaneous and continuous periodic motion of a system driven by stable external excitation [3]. The system is a typical non-equilibrium thermodynamic process [4,5], where system energy is dynamic due to energy dissipation [6] caused by internal resistance. During the motion process, the system encounters environmental stimuli, prompting a response. Utilizing its own material properties, the system gathers energy from the surroundings and transforms it into internal kinetic energy, keeping its parts in constant motion [7,8]. When the energy absorbed from environmental stimuli perfectly compensates the energy lost to resistance during motion, the system achieves a stable state of continuous motion. Additionally, the system’s internal energy undergoes periodic amplification via external excitation, enabling self-sustained motion at a constant frequency [9,10]. Remarkably, the amplitude and period of the system’s new steady state are independent of its initial state, solely governed by inherent properties, demonstrating robust stability [11,12]. Moreover, during the process of achieving periodic motion, the system does not require a sophisticated controller to regulate its movement [13]. Instead, it relies on its own internal feedback mechanism [14] to generate spontaneous motion. Frequent feedback mechanisms include the synchronization of massive deformations and chemical reactions [15], the self-shading mechanism [16], the concerted integration of droplet evaporation and membrane deformation processes [17], the coupling of air expansion with liquid column movement [18], the coupling of plate bending with chemical reactions [19], photothermal surface tension gradients [20], and water exchange and thin film deflection intertwined in multiple processes [21,22].

Based on the characteristics of self-sustained oscillation [23,24], researchers have leveraged its advantages and applied it in various fields, such as micro-autonomous robots [25,26], nanogenerators [27], micro- and nanodevices [28], medical devices [29], energy harvesting [30,31], active machines [32,33,34], autonomous separators [35], and mass transport devices [36]. In particular, harvesting vibrational energy from bridges and roads for wireless sensors faces challenges due to its weakness and inconsistency. Light-driven self-oscillating systems [37] convert light into mechanical energy, enabling a continuous power supply for monitoring through mechanical-to-electrical conversion [38,39]. This efficient clean energy approach holds great potential.

Achieving self-oscillation requires incorporating responsive materials within the system’s components to environmental stimuli, including temperature [40], light [41], magnetic [42], electricity [43,44], and humidity [45]. The corresponding environmentally responsive materials contain soft magnetic polymers [46], ionogels [47], liquid crystal elastomers [48,49], shape memory polymers [50], and smart polymer hydrogels [51]. Liquid crystal elastomers (LCEs) [52,53] transition between anisotropic and isotropic states under stimuli, achieving reversible deformations [54,55]. Recent advancements have made them programmable and high-energy dense materials. Currently, LCE materials have been widely applied in various fields such as soft robotics [56,57], actuators [58], optics [59], wearable electronics [60], and medication delivery [61]. Based on the characteristics and advantages of LCE’s stimulus response, researchers have developed multiple self-oscillation modes, including vibration [62,63], buckling [64], self-galloping [65], chaos [66,67], sit-ups of LCE thin film [22], eversion or inversion [68,69] and the coordinated motion [70] of multiple coupled oscillators. Simultaneously, self-oscillating machines based on LCEs involve multiple aspects and demonstrate significant application potential and value, such as in thin film motors [71], cantilevered oil paper [72], perpetual motion machines [73], self-paddling boats [74], engines [75], self-moving automobiles [76], and other fields.

Given the combined interest in light, a clean, stable, and sustainable energy source, and LCEs, utilizing photochemistry for reversible deformations and rapid responses [77,78] has consistently attracted significant research attention. As the optical-mechanical self-oscillation systems of these materials are meticulously explored, a critical observation emerges: trajectory variations in rotational systems [79,80], prevalent in both nature and engineering domains, exhibit dynamic balance and stability, revealing fundamental physics. Despite extensive research on the self-oscillating mechanisms of light-driven LCE-slider systems, a persistent challenge lies in the restriction of system movements solely to circular trajectory. To overcome this limitation and more accurately simulate intricate natural movements, we introduce an innovative light-powered LCE fiber-slider system that is uniquely designed to enable continuous self-circling along an elliptical track. The main content of the article is as follows. In Section 2, we construct a theoretical model for the deformation of LCE fibers subjected to optical stimulation, grounded in the theories of deformation under environmental stimuli and opto-mechanical coupling dynamics. Furthermore, we deduce a nonlinear dynamic model that accurately portrays the elliptical trajectory motion exhibited by the system under the influence of light. In Section 3, we thoroughly explore two modes of system motion—static and self-circling—induced by a strategically designed illuminated area. Furthermore, we analyze the operational mechanism of self-circling based on opto-mechanical coupling and delve into the nonlinear dynamic behaviors inherent to the system. In Section 4, we apply the mechanical model and numerical methods to examine the effects of parameter variations on system performance. Additionally, we analyze the relationship between trajectory alterations and self-circling, conducting a rigorous quantitative assessment. In Section 5, a concise overview of the conclusions of this paper is presented.

## 2. Theoretical Model and Formulation

First, we introduce a newly proposed nonlinear dynamic response system capable of continuously self-circling under light stimulation that is composed of a light-powered LCE fiber, a slider, and a rigid elliptical track. Subsequently, based on Newton’s second law and the nonlinear dynamics model of light-powered LCE fiber, we delve into analyzing the dynamic force variations and movement patterns during the system’s operation, alongside performing geometric parameter calculations. In addition, we derive a nonlinear dynamic response model for the system’s self-sustaining periodic motion. Lastly, to eliminate the influence of dimension among parameters in the control equation, we normalize the parameters through the process of nondimensionalization. Ultimately, numerical analytical calculations yield the dimensionless motion control equation of the system.

### 2.1. Dynamics of Self-Circling System

**Figure 1:**
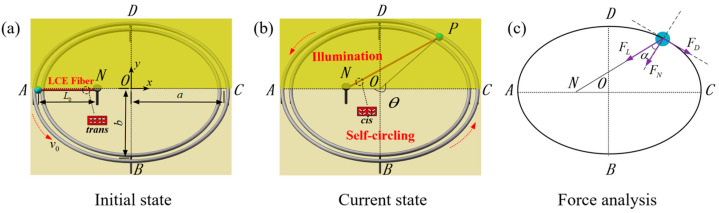
Diagram of a light-powered self-circling device on a plane including the LCE fiber, sliding element, and fixed elliptical track: (**a**) Initial state; (**b**) Current state; and (**c**) Force analysis. Under constant light exposure, the slider equipped with the LCE fiber achieves autonomous, sustained, and periodic movement along the elliptical track.

Figure 1 and Video S1 present the two-dimensional structural schematic of a light-powered self-circling LCE-slider system, including an LCE fiber energized by light, a sliding block, and a stationary elliptical track. Specifically, one end of the LCE fiber is linked to a slider weighing m, and the other end is fastened to point *N*, which is located on the major axis of the elliptical track. Given the LCE fiber’s substantially lighter mass compared to the slider, we disregard its impact on the motion. The slider is securely mounted on the elliptical track and is capable of performing a stable, periodic self-circling motion along an elliptical trajectory, initiated by a predefined initial velocity $v_{0}$ and under specific illumination conditions. Designating the centroid of the elliptical track as the origin (*O*), we establish the major axis along the *x*-axis and the minor axis along the *y*-axis. Points *A* and *C* are positioned at the opposite ends of the major axis, marking the extremities, while points *B* and *D* occupy the extremities of the minor axis on the ellipse. In addition, we designate point *A* as the starting position of the self-circling slider, where the rotating angular displacement θ is zero. In the depiction of the system as shown in Figure 1a, the LCE fiber is represented as being unstressed and having an initial length of $L_{0}$.

The illuminated zone is indicated by the yellow area in Figure 1a,b, whereas the non-illuminated zone is represented by the remaining half of the lighter colored region. Under the stimulation of the illuminated zone, the system exhibits two motion modes: static and self-circling. Initiating from point *A*, the slider moves counterclockwise due to inertia, decelerating under the influence of resistance and the reverse pulling force from the LCE. Upon entering the illuminated zone, the LCE fibers undergo a photochemical reaction, transforming the photosensitive groups from a stable *trans-isomer* to a *cis-isomer*, resulting in nonlinear contraction in a predetermined direction. This contraction accelerates the slider, propelling it forward and regulating its speed as it enters the illuminated zone each time. Exiting the illuminated zone, the LCE fibers revert to their stable trans-isomer configuration due to their reversible phase transition, causing the slider to decelerate once again under inertia. This process repeats itself in a periodic fashion, with the slider’s entry speed into the illuminated zone gradually stabilizing and maintaining this motion state for subsequent rotations. From an energy perspective, as the slider shuttles between the illuminated and non-illuminated zones, the system’s energy periodically amplifies and converts into mechanical energy, while simultaneously dissipating energy due to resistance. After multiple cycles of energy conversion, a new steady state is established when the energy absorbed by the system from the illuminated zone balances the energy dissipated by resistance.

Figure 1c depicts the force diagram pertaining to the slider of the system. Given that point *A* serves as the initial point for the rotation of the slider and that the angular displacement θ of rotation is measured from *OA* as the starting edge, the elliptical equation within the plane *xoy*, as the slider rotates counterclockwise, is given as x=−acosθ, y=−bsinθ, where a and b represent the semi-major and semi-minor axes of the ellipse, respectively. In the course of the slider circling the elliptical trajectory, the primary research emphasis lies in the detailed analysis of the magnitude and fluctuations of its tangential velocity. Based on Newton’s second law [81], when θ falls within the interval from 2kπ to $\left(2k+1\right)\pi$, where k is a natural number, the tangential mechanical control equation of the slider’s motion is as shown below:

(1) $$m\frac{dv_{\tau }}{dt}=-F_{L}\sin\alpha -F_{D},$$

where $\frac{dv_{\tau }}{dt}\;$represents the tangential acceleration of the slider at its instantaneous position, $F_{L}$ is the tensile force exerted by the LCE fiber on the slider, and $F_{D}$ denotes the total damping force.

When θ lies between $\left(2k+1\right)\pi$ and $\left(2k+2\right)\pi$, where k is a natural number, the mechanical control equation is given as follows:

(2) $$m\frac{dv_{\tau }}{dt}=F_{L}\sin\alpha -F_{D},$$

where the explanations of the variables $\frac{dv_{\tau }}{dt}$, $F_{L}$, and $F_{D}$ in Equation (2) are the same as above in Equation (1). According to the angle formula in the structure shown in Figure 1c, we can derive that $\sin\alpha =\frac{\left|\sin\theta \left(a\left(a-L_{0}\right)+\left(b^{2}-a^{2}\right)\cos\theta \right)\right|}{\sqrt{{\left(\sin\theta \right)}^{2}{\left[a\left(a-L_{0}\right)+\left(b^{2}-a^{2}\right)\cos\theta \right]}^{2}+b^{2}{\left[\left(a-L_{0}\right)\cos\theta -a\right]}^{2}}}$, where α represents the angle formed between the LCE string and the normal direction at any given point on the ellipse.

Given the equations x=−acosθ and y=−bsinθ, the velocity of the slider along the tangential direction can be expressed as follows:

(3) $$v_{\tau }=\dot{\theta }\sqrt{a^{2}{\left(\sin\theta \right)}^{2}+b^{2}{\left(\cos\theta \right)}^{2}.}$$

By differentiating Equation (3) with respect to time, we can derive the tangential acceleration of the slider as follows:

(4) $$\frac{dv_{\tau }}{dt}=\ddot{\theta }\sqrt{a^{2}{\left(\sin\theta \right)}^{2}+b^{2}{\left(\cos\theta \right)}^{2}}+{\left(\dot{\theta }\right)}^{2}\frac{\left(a^{2}-b^{2}\right)\sin\theta \cos\theta }{\sqrt{a^{2}{\left(\sin\theta \right)}^{2}+b^{2}{\left(\cos\theta \right)}^{2}}}.$$

The tension exerted by the LCE fiber on the slider satisfies Hooke’s law, exhibiting a linear relationship with elastic deformation, which can be expressed as follows [82]:

(5) $$F_{L}=KL_{0}\varepsilon_{e}\left(t\right),$$

where K is the elastic coefficient of the LCE fiber, $\varepsilon_{e}\left(t\right)$ denotes the elastic strain in the LCE fiber, and $L_{0}$ is the initial length of the LCE fiber. Given the contraction characteristics of LCE under light stimulation and based on the assumption of small deformations, the elastic strain $\varepsilon_{e}\left(t\right)$ of the LCE fiber can be expressed as the difference between the total strain $\varepsilon_{tot}\left(t\right)$ and the light-induced contraction strain $\varepsilon_{L}\left(t\right)$ under light stimulation. Therefore, Equation (5) can be rewritten as follows:

(6) $$F_{L}=KL_{0}\left(\varepsilon_{tot}\left(t\right)-\varepsilon_{L}\left(t\right)\right).$$

When illuminated, the LCE experiences unidirectional nonlinear contraction. For brevity, let $\varepsilon_{tot}\left(t\right)$ denote the instantaneous elongation ratio of the LCE, specifically, $\varepsilon_{tot}\left(t\right)=\frac{L-L_{0}}{L_{0}}$. By incorporating this into Equation (6), we can obtain the following:

(7) $$F_{L}=K\left(L-L_{0}-L_{0}\varepsilon_{L}\left(t\right)\right),$$

where L signifies the current length of the LCE fiber, and its value may be obtained by $L=\sqrt{{\left(a-L_{0}\right)}^{2}+a^{2}{\left(\cos\theta \right)}^{2}+b^{2}{\left(\sin\theta \right)}^{2}-2\left(a-L_{0}\right)\cos\theta \sqrt{a^{2}{\left(\cos\theta \right)}^{2}+b^{2}{\left(\sin\theta \right)}^{2}}}$.

The damping force experienced by the slider during its motion is nonlinear, and it is assumed to have a quadratic relationship with the tangential velocity, which can be expressed as follows:

(8) $$F_{D}=\beta_{1}v_{\tau }+\beta_{2}{v_{\tau }}^{2},$$

where $\beta_{1}$ and $\beta_{2}$ represent the damping coefficients related to the medium.

By substituting Equations (3), (4), (7) and (8), and relevant geometric parameters into Equations (1) and (2), we can obtain the following for the segment $\left(2k\pi \right.,\left(2k+1\right)\left.\pi \right)$, where k is a natural number:

(9) $$\begin{array}{l} m\left(\ddot{\theta }\sqrt{a^{2}{\left(\sin\theta \right)}^{2}+b^{2}{\left(\cos\theta \right)}^{2}}+{\left(\dot{\theta }\right)}^{2}\frac{\left(a^{2}-b^{2}\right)\sin\theta \cos\theta }{\sqrt{a^{2}{\left(\sin\theta \right)}^{2}+b^{2}{\left(\cos\theta \right)}^{2}}}\right)\\ \;=-K\left(L-L_{0}-L_{0}\varepsilon_{L}\left(t\right)\right)\frac{\left|\sin\theta \left(a\left(a-L_{0}\right)+\left(b^{2}-a^{2}\right)\cos\theta \right)\right|}{\sqrt{{\left(\sin\theta \right)}^{2}{\left[a\left(a-L_{0}\right)+\left(b^{2}-a^{2}\right)\cos\theta \right]}^{2}+b^{2}{\left[\left(a-L_{0}\right)\cos\theta -a\right]}^{2}}}\\ \;-\beta_{1}\dot{\theta }\sqrt{a^{2}{\left(\sin\theta \right)}^{2}+b^{2}{\left(\cos\theta \right)}^{2}}-\beta_{2}{\left(\dot{\theta }\sqrt{a^{2}{\left(\sin\theta \right)}^{2}+b^{2}{\left(\cos\theta \right)}^{2}}\right)}^{2}. \end{array}$$

For the segment $\left(\left(2k+1\right)\pi \right.,\left.\left(2k+2\right)\pi \right)$, where k is a natural number

(10) $$m\left(\ddot{\theta }\sqrt{a^{2}{\left(\sin\theta \right)}^{2}+b^{2}{\left(\cos\theta \right)}^{2}}+{\left(\dot{\theta }\right)}^{2}\frac{\left(a^{2}-b^{2}\right)\sin\theta \cos\theta }{\sqrt{a^{2}{\left(\sin\theta \right)}^{2}+b^{2}{\left(\cos\theta \right)}^{2}}}\right)=K\left(L-L_{0}-\;L_{0}\varepsilon_{L}\left(t\right)\right)\frac{\left|\sin\theta \left(a\left(a-L_{0}\right)+\left(b^{2}-a^{2}\right)\cos\theta \right)\right|}{\sqrt{{\left(\sin\theta \right)}^{2}{\left[a\left(a-L_{0}\right)+\left(b^{2}-a^{2}\right)\cos\theta \right]}^{2}+b^{2}{\left[\left(a-L_{0}\right)\cos\theta -a\right]}^{2}}}-\beta_{1}\dot{\theta }\sqrt{a^{2}{\left(\sin\theta \right)}^{2}+b^{2}{\left(\cos\theta \right)}^{2}}-\;\beta_{2}{\left(\dot{\theta }\sqrt{a^{2}{\left(\sin\theta \right)}^{2}+b^{2}{\left(\cos\theta \right)}^{2}}\right)}^{2}.$$

### 2.2. Dynamic Model of LCE Fiber

This section explores the underlying mechanism of the contractile strain response in LCE fibers under light stimulation. Under small deformation conditions, the contractile strain of LCE fibers exhibits a clear linear relationship with the number fraction of their internal *cis-isomers*. The contractile strain can be determined as follows:

(11) $$\varepsilon_{L}\left(t\right)=-C\varphi \left(t\right),$$

where C represents the contraction coefficient of the LCE fiber.

When photosensitive molecules in LCE fibers encounter light, they rearrange, leading to the *trans-cis* isomerization of the LCE fibers, which in turn causes a macroscopic volume contraction phenomenon. Yu et al. [83] found that azobenzene molecules in LCE fibers can selectively absorb light in a specific direction, exhibiting a strong absorption effect for ultraviolet light with a wavelength of around 360 nm. This allows for efficient conversion of light energy into mechanical energy. Furthermore, the reversible deformation of LCE fibers does not exhibit fatigue, meaning that the system can achieve a cyclic and repetitive mechanism of opto-mechanical coupling. Notably, the number fraction of cis isomers in LCE fibers increases when they enter a light-exposed area and decreases to zero after leaving the light-exposed area. This indicates that the degree of strain contraction in LCE fibers can be characterized by the number fraction of cis isomers. Additionally, considering that the *cis-trans* isomerization in LCE is not affected by strain, we can neglect this factor. Previous studies [84,85] have shown that the number fraction of *cis isomers* is influenced by three factors: thermally activated transition from *trans* to *cis*, thermally driven relaxation from *cis* back to *trans*, and photo-driven promotion of the *trans*-to-*cis* isomerization. Compared to the other two factors, the influence of thermal activation on the number fraction of *cis isomers* is negligible, so we disregard the impact of thermal activation. Based on the above, the governing equation for the number fraction of *cis isomers*, expressed in differential form, is as follows:

(12) $$\frac{\partial \varphi \left(t\right)}{\partial t}=\eta_{0}I\left(1-\varphi \left(t\right)\right)-\frac{\varphi \left(t\right)}{T_{0}},$$

where $\eta_{0}$ denotes the light absorption constant, I refers to the light intensity, and $T_{0}$ represents the thermally driven relaxation time from *cis* to *trans*. By solving Equation (12), we can obtain the following:

(13) $$\varphi \left(t\right)=\frac{\eta_{0}T_{0}I}{\eta_{0}T_{0}I+1}+\left(\varphi_{0}-\frac{\eta_{0}T_{0}I}{\eta_{0}T_{0}I+1}\right)exp\left[-\frac{t}{T_{0}}\left(\eta_{0}T_{0}I+1\right)\right],$$

where $\varphi_{0}$ denotes the initial number fraction of *cis isomers* for the photosensitive molecules in non-illuminated areas.

Assuming that the initial value of $\varphi_{0}$ for the number fraction of *cis isomers* is zero when entering the illuminated area, Equation (13) can be simplified as follows:

(14) $$\varphi \left(t\right)=\frac{\eta_{0}T_{0}I}{\eta_{0}T_{0}I+1}\left(1-exp\left[-\frac{t}{T_{0}}\left(\eta_{0}T_{0}I+1\right)\right]\right).$$

In non-illuminated area, the value of I is 0. Substituting this into Equation (14) results in the following:

(15) $$\varphi \left(t\right)=\varphi_{0}exp\left(-\frac{t}{T_{0}}\right).$$

For Equation (12), when t=0, $\varphi_{0}$ reaches its maximum value, which is denoted as $\varphi_{0max}=\frac{\eta_{0}T_{0}I}{\eta_{0}T_{0}I+1}$. Substituting this into Equation (15) yields the following:

(16) $$\varphi \left(t\right)=\frac{\eta_{0}T_{0}I}{\eta_{0}T_{0}I+1}exp\left(-\frac{t}{T_{0}}\right).$$

### 2.3. Nondimensionalization

To simplify the equation and directly study the impact of various parameters on the system, dimensionless parameters are now introduced as follows: $\bar{\dot{\theta }}=\dot{\theta }T_{0},$ $\bar{\ddot{\theta }}=\ddot{\theta }T_{0}^{2},$ $\bar{t}=t/T_{0},$ $\bar{K}=KT_{0}^{2}/m,$ $\bar{I}=\eta_{0}T_{0}I_{0},$ $\bar{\beta_{1}}=\beta_{1}T_{0}/m,$ $\bar{\beta_{2}}=\frac{\beta_{2}T_{0}^{2}}{m},$ $\bar{\varphi }\left(t\right)=\varphi \left(t\right)\frac{\eta_{0}T_{0}I+1}{\eta_{0}T_{0}I},$ $\bar{a}=a/L_{0},\;$and $\bar{b}=b/L_{0}$. Substituting the dimensionless parameters mentioned above into Equations (9) and (10), the non-dimensionalized form of the nonlinear mechanical control equation for the slider along the tangential direction can be obtained as follows for the segment $\left(2k\pi \right.,\left(2k+1\right)\left.\pi \right)$, where k is a natural number:

(17) $$\bar{\ddot{\theta }}=-\bar{K}\frac{\sqrt{{\left(\bar{a}-1\right)}^{2}+{\bar{a}}^{2}{\left(\cos\theta \right)}^{2}+{\bar{b}}^{2}{\left(\sin\theta \right)}^{2}-2\left(\bar{a}-1\right)\cos\theta \sqrt{\bar{a}{\left(\cos\theta \right)}^{2}+{\bar{b}}^{2}{\left(\sin\theta \right)}^{2}}}-1-\varepsilon_{L}\left(t\right)}{\sqrt{{\bar{a}}^{2}{\left(\sin\theta \right)}^{2}+{\bar{b}}^{2}{\left(\cos\theta \right)}^{2}}}\frac{\left|\sin\theta \left(\bar{a}\left(\bar{a}-1\right)+\left({\bar{b}}^{2}-{\bar{a}}^{2}\right)\cos\theta \right)\right|}{\sqrt{{\left(\sin\theta \right)}^{2}{\left[\bar{a}\left(\bar{a}-1\right)+\left({\bar{b}}^{2}-{\bar{a}}^{2}\right)\cos\theta \right]}^{2}+{\bar{b}}^{2}{\left[\left(\bar{a}-1\right)\cos\theta -\bar{a}\right]}^{2}}}-\bar{\beta_{1}}\bar{\dot{\theta }}-\bar{\beta_{2}}{\left(\bar{\dot{\theta }}\right)}^{2}\sqrt{{\left(\sin\theta \right)}^{2}+{\left(\frac{\bar{b}}{\bar{a}}\right)}^{2}{\left(\cos\theta \right)}^{2}}-\;{\left(\bar{\dot{\theta }}\right)}^{2}\frac{\left({\bar{a}}^{2}-{\bar{b}}^{2}\right)\sin\theta \cos\theta }{{\bar{a}}^{2}{\left(\sin\theta \right)}^{2}+{\bar{b}}^{2}{\left(\cos\theta \right)}^{2}}.$$

For the segment $\left(\left(2k+1\right)\pi \right.,\left.\left(2k+2\right)\pi \right)$, where k is a natural number, the following is obtained:

(18) $$\bar{\ddot{\theta }}=\bar{K}\frac{\sqrt{{\left(\bar{a}-1\right)}^{2}+{\bar{a}}^{2}{\left(\cos\theta \right)}^{2}+{\bar{b}}^{2}{\left(\sin\theta \right)}^{2}-2\left(\bar{a}-1\right)\cos\theta \sqrt{\bar{a}{\left(\cos\theta \right)}^{2}+{\bar{b}}^{2}{\left(\sin\theta \right)}^{2}}}-1-\varepsilon_{L}\left(t\right)}{\sqrt{{\bar{a}}^{2}{\left(\sin\theta \right)}^{2}+{\bar{b}}^{2}{\left(\cos\theta \right)}^{2}}}\frac{\left|\sin\theta \left(\bar{a}\left(\bar{a}-1\right)+\left({\bar{b}}^{2}-{\bar{a}}^{2}\right)\cos\theta \right)\right|}{\sqrt{{\left(\sin\theta \right)}^{2}{\left[\bar{a}\left(\bar{a}-1\right)+\left({\bar{b}}^{2}-{\bar{a}}^{2}\right)\cos\theta \right]}^{2}+{\bar{b}}^{2}{\left[\left(\bar{a}-1\right)\cos\theta -\bar{a}\right]}^{2}}}-\bar{\beta_{1}}\bar{\dot{\theta }}-\;\bar{\beta_{2}}{\left(\bar{\dot{\theta }}\right)}^{2}\sqrt{{\left(\sin\theta \right)}^{2}+{\left(\frac{\bar{b}}{\bar{a}}\right)}^{2}{\left(\cos\theta \right)}^{2}}-{\left(\bar{\dot{\theta }}\right)}^{2}\frac{\left({\bar{a}}^{2}-{\bar{b}}^{2}\right)\sin\theta \cos\theta }{{\bar{a}}^{2}{\left(\sin\theta \right)}^{2}+{\bar{b}}^{2}{\left(\cos\theta \right)}^{2}}.$$

Substituting into Equation (14), the dimensionless form of the number fraction of *cis isomers* in the illuminated area is expressed as follows:

(19) $$\bar{\varphi }\left(t\right)=1-exp\left[-\left(1+\bar{I}\right)\bar{t}\right].$$

Substituting into Equation (16), the dimensionless form of the number fraction of *cis isomers* in the non-illuminated area is expressed as follows:

(20) $$\bar{\varphi }\left(t\right)=exp\left(-\bar{t}\right).$$

The non-dimensionalized form of the tangential component $\bar{F_{L\tau }}$ of the tensile force exerted by the LCE fiber on the slider can be expressed as noted below.

When θ ranges from 2kπ to $\left(2k+1\right)\pi$, we can derive Equation (21) as follows:

(21) $$\bar{F_{L\tau }}=-\bar{K}\frac{\sqrt{{\left(\bar{a}-1\right)}^{2}+{\bar{a}}^{2}{\left(\cos\theta \right)}^{2}+{\bar{b}}^{2}{\left(\sin\theta \right)}^{2}-2\left(\bar{a}-1\right)\cos\theta \sqrt{\bar{a}{\left(\cos\theta \right)}^{2}+{\bar{b}}^{2}{\left(\sin\theta \right)}^{2}}}-1-\varepsilon_{L}\left(t\right)}{\sqrt{{\bar{a}}^{2}{\left(\sin\theta \right)}^{2}+{\bar{b}}^{2}{\left(\cos\theta \right)}^{2}}}\frac{\left|\sin\theta \left(\bar{a}\left(\bar{a}-1\right)+\left({\bar{b}}^{2}-{\bar{a}}^{2}\right)\cos\theta \right)\right|}{\sqrt{{\left(\sin\theta \right)}^{2}{\left[\bar{a}\left(\bar{a}-1\right)+\left({\bar{b}}^{2}-{\bar{a}}^{2}\right)\cos\theta \right]}^{2}+{\bar{b}}^{2}{\left[\left(\bar{a}-1\right)\cos\theta -\bar{a}\right]}^{2}}}$$

When θ ranges from $\left(2k+1\right)\pi$ to $\left(2k+2\right)\pi$, we can derive Equation (22) as follows:

(22) $$\bar{F_{L\tau }}=\bar{K}\frac{\sqrt{{\left(\bar{a}-1\right)}^{2}+{\bar{a}}^{2}{\left(\cos\theta \right)}^{2}+{\bar{b}}^{2}{\left(\sin\theta \right)}^{2}-2\left(\bar{a}-1\right)\cos\theta \sqrt{\bar{a}{\left(\cos\theta \right)}^{2}+{\bar{b}}^{2}{\left(\sin\theta \right)}^{2}}}-1-\varepsilon_{L}\left(t\right)}{\sqrt{{\bar{a}}^{2}{\left(\sin\theta \right)}^{2}+{\bar{b}}^{2}{\left(\cos\theta \right)}^{2}}}\frac{\left|\sin\theta \left(\bar{a}\left(\bar{a}-1\right)+\left({\bar{b}}^{2}-{\bar{a}}^{2}\right)\cos\theta \right)\right|}{\sqrt{{\left(\sin\theta \right)}^{2}{\left[\bar{a}\left(\bar{a}-1\right)+\left({\bar{b}}^{2}-{\bar{a}}^{2}\right)\cos\theta \right]}^{2}+{\bar{b}}^{2}{\left[\left(\bar{a}-1\right)\cos\theta -\bar{a}\right]}^{2}}}$$

From Equations (17) and (18), it can be seen that the mechanical control equation of the system is a second-order nonlinear differential equation, which makes it difficult to obtain an exact solution directly. Therefore, we utilize the iterative concept in numerical analysis and employ the fourth-order Runge–Kutta numerical method for iterations. We conduct numerical calculations and analysis using MATLAB R2021a software. By adjusting the parameters in the program, such as $\bar{I}$, C, $\bar{K}$, $\bar{v_{0}}$, $\bar{\beta_{1}}$, $\bar{\beta_{2}}$, $\bar{a}$, and $\bar{b}$, the system can achieve self-sustained periodic circling. At the same time, we investigate the impact of the ratio of the major and minor axes of the ellipse on the trajectory motion. Additionally, we can also obtain the number fraction of *cis isomers*, tension, resistance, contractile strain, and angular velocity at instantaneous positions.

## 3. Two Dynamic Regimes and Mechanism of Self-Circling

In this section, we conduct a numerical analysis of the derived mechanical control equations to study the motion response of the light-responsive LCE-slider. First, two motion regimes of the system under constant illumination are described. Then, the fundamental principles and operational mechanisms of the self-circling are explored in greater depth.

### 3.1. Two Motion Regimes

To study the self-circling characteristics of the LCE-slider system, it is first necessary to obtain the dimensionless range of system parameters. Based on previous experimental studies and validations [86,87], the actual values of relevant material and structural parameters are provided in Table 1. In this case, the corresponding dimensionless range of these parameters is given in Table 2.

**Figure 2:**
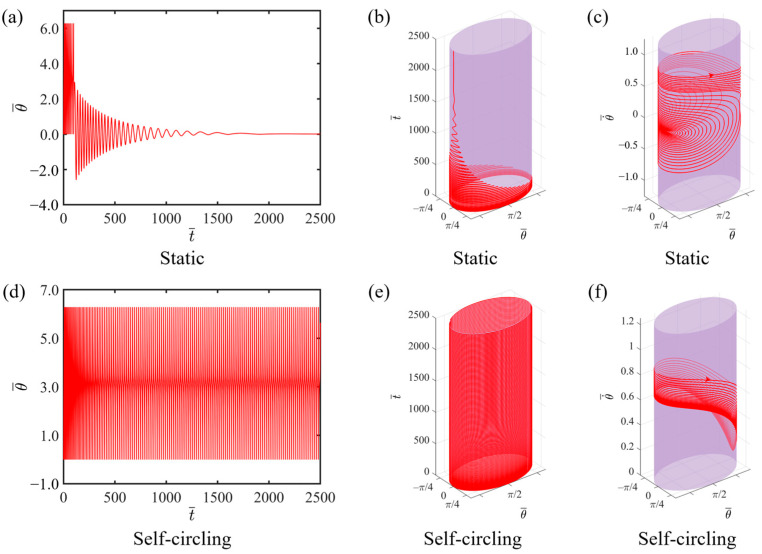
Two motion regimes of the system under continuous light exposure: static state and self-circling state. (**a**,**b**) Time-dependent diagram of angular displacement at $\overset{-}{I}=0.1$; (**c**) Angular velocity dependence on angular displacement at $\overset{-}{I}=0.1$; (**d**,**e**) Time-dependent diagram of angular displacement at $\overset{-}{I}=0.3$; (**f**) Angular velocity dependence on angular displacement $\overset{-}{I}=0.3$.

By numerically solving Equations (17) and (18), we can obtain the time-dependent diagram of angular displacement and the angular velocity dependence on angular displacement of the LCE-slider system. In this study, the relevant parameter settings for the numerical calculations are as follows: C=0.3, $\bar{K}=1.0$, $\bar{v_{0}}=0.9$, $\theta_{0}=\left[\pi ,2\pi \right]$, $\bar{\beta_{1}}=0.007$, $\bar{\beta_{2}}=0.0006$, $\bar{a}=2.0$, and $\bar{b}=1.9$. The results indicate that the system exhibits two motion regimes: a static state and a self-circling state with light intensity $\bar{I}\;$values of 0.1 and 0.3, respectively. Figure 2a,b depict the two-dimensional and three-dimensional time-dependent diagrams of angular displacement, respectively, when $\overset{-}{I}=0.1$. From the corresponding three-dimensional phase trajectory diagram, it can be observed that due to resistance, the angular velocity gradually decreases as the rotation progresses, ultimately leading to the phase trajectory converging to a single point and signifying the attainment of a static state, as shown in Figure 2c. Figure 2d,e present time-dependent diagrams of angular displacement when $\overset{-}{I}=0.3$. After completing a period of circular motion, the circling frequency of the system gradually becomes constant, and the system continues to rotate periodically at this frequency, indicating that it has reached a self-circling state. As the number of rotations increases, the angular velocity gradually stabilizes and maintains the same pattern of change for each subsequent rotation, repeating a consistent pattern for every cycle, as illustrated in Figure 2f. The reason for the occurrence of self-circling is that the system gains sufficient energy, which can precisely compensate for the energy dissipated during the system’s motion. A detailed explanation is provided in the following section.

### 3.2. Mechanism of Self-Circling

**Figure 3:**
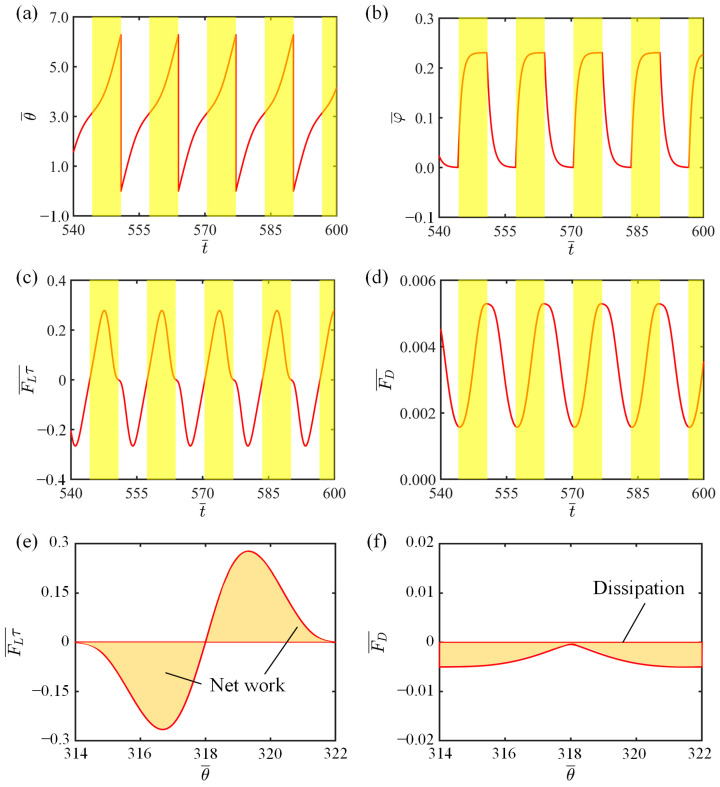
Self-circling mechanism of the system. (**a**) Time-dependent curve of angular displacement; (**b**) Time-dependent curve of the number fraction of *cis*-*isomers* in the LCE fiber; (**c**) Time-dependent curve of tangential tension in the LCE fiber; (**d**) Time-dependent curve of damping force; (**e**) Variation of tangential tension in the LCE fiber with respect to the angular displacement; (**f**) Variation of damping force with respect to angular displacement. The yellow squares in the figure denote illuminated region.

In this section, we elaborate on the operation mechanism of the LCE-slider system’s spontaneous circling under constant illumination. For this purpose, the variation curves of key physical quantities are plotted in Figure 3. Some of the dimensionless parameters of the system are set as follows: $\overset{-}{I}=0.3$, C=0.3, $\bar{K}=1.0$, $\bar{v_{0}}=0.9$, $\theta_{0}=\left[\pi ,2\pi \right]$, $\bar{\beta_{1}}=0.007$, $\bar{\beta_{2}}=0.0006$, $\bar{a}=2.0$, and $\bar{b}=1.9$. Figure 3a depicts the change in the angular displacement of the slider over time during the movement, with the yellow striped area representing the illuminated region. It can be observed that the slider continuously travels between the illuminated and non-illuminated regions with a stable period. Figure 3b exhibits a periodic variation in the number fraction of the *cis-isomer* in the LCE material. As the LCE string enters the illuminated region, the number fraction of the *cis-isomer* in the LCE fiber gradually increases and reaches a peak. Upon exiting the illuminated region, the number fraction of the *cis-isomer* in the LCE fiber rapidly decreases to zero. Figure 3c,d shows the temporal variations of the tangential tension in the LCE fiber and the resistance encountered by the slider, respectively. Under the influence of illumination, the LCE fiber undergoes a unidirectional contraction, leading to an increase in the tension of the LCE string. However, upon exiting the illuminated region, due to the reversibility of the LCEs’ photoresponsive contraction, the photoresponsive contraction recovers, resulting in a decrease in the tension of the LCE string. As the slider circles periodically, the tangential tension of the LCE also varies periodically. The resistance encountered by the slider during its movement is related to its tangential velocity. In the non-illuminated region, the slider’s velocity decreases; however, in the illuminated region, its velocity increases. Therefore, from the non-illuminated region to the illuminated region, the resistance first decreases and then increases, exhibiting a periodic variation.

Furthermore, Figure 3e illustrates the relationship curve between the tangential tension of the LCE fiber and the angular displacement, and the area enclosed by the hysteretic loop represents the energy absorbed by the system from the illuminated region during one circling cycle. Numerical analysis calculates that the area enclosed by the hysteretic loop, which represents the net work, is 0.445. Figure 3f depicts the variation of the damping force with the angular displacement. The area enclosed by the closed curve represents the negative work done by the resistance in a single cycle, i.e., the energy consumed by the system during a single period. The numerically calculated value is also approximately 0.445. This indicates that the energy dissipated by the system during its motion can be precisely compensated by the energy absorbed from the illuminated region, which is the energy compensation mechanism that enables the system’s spontaneous circle, namely, the circling mechanism of the LCE-slider system.

## 4. Parameter Study

Based on a profound understanding of the two motion states and their internal mechanisms, this section focuses on studying the specific regulatory effects of key external parameters including $\overset{-}{I}$, C, $\bar{K}$, $\bar{v_{0}}$, $\bar{\beta_{1}}$, $\bar{\beta_{2}}$, $\overset{-}{a},\;$and $\overset{-}{b}$ on the angular velocity and frequency of the LCE-slider system, conducting a detailed and exhaustive quantitative analysis.

### 4.1. Effect of Light Intensity

**Figure 4:**
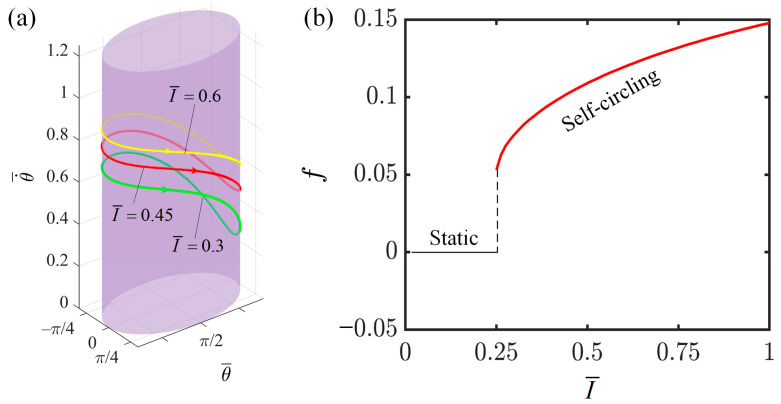
The effect of light intensity on the self-circling frequency. (**a**) Depictions of limit cycles at $\overset{-}{I}=0.3$, 0.45 and 0.6. (**b**) Self-circling frequency variations with light intensities.

This section investigates the impact of light intensity on the angular velocity and frequency of the LCE-slider system. First, we set the other parameters as follows: C=0.3, $\bar{K}=1.0$, $\bar{v_{0}}=0.9$, $\bar{\beta_{1}}=0.007$, $\bar{\beta_{2}}=0.0006$, $\bar{a}=2.0$, and $\bar{b}=1.9$. With a stable and continuous illumination maintained within the range of π to 2π, we analyze how variations in light intensity affect the motion patterns and states of the LCE-slider system. As can be seen from Figure 4a, when I=0.6, the limit cycle is higher than that at I=0.3 and I=0.45, indicating that an increase in light intensity accelerates the rotation speed of the slider. Figure 4b illustrates the relationship between light intensity and the motion frequency of the slider. When the light intensity is below 0.25, the LCE fibers do not absorb sufficient energy in the illuminated region to maintain the energy consumption during the slider’s motion, resulting in the system remaining in the static state. However, when the light intensity exceeds 0.25, the LCE fibers are able to absorb adequate energy to compensate for the energy dissipated during the slider’s motion, enabling the system to maintain continuous rotation. In this scenario, the system is in a state of self-circling. Additionally, we can observe that as the light intensity increases, the motion frequency of the slider exhibits a nonlinear increase. This is attributed to the fact that an increase in light intensity leads to a greater degree of photo-contraction in the LCE fibers. The larger this contraction is, the greater the net work done by the LCE fibers on the slider, resulting in more kinetic energy being imparted to the slider. Therefore, the instantaneous angular velocity of the slider’s motion increases, the motion period shortens, and the motion frequency increases. This finding provides insights into enhancing the operational efficiency of this system in engineering applications.

### 4.2. Effect of Contraction Coefficient of LCE

**Figure 5:**
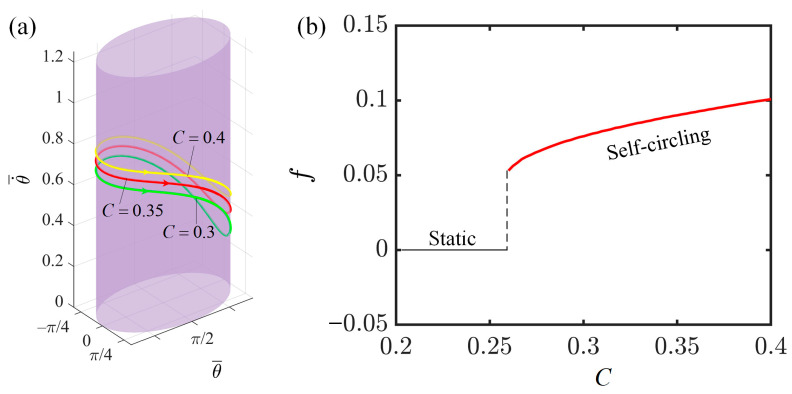
The effect of the contraction coefficient on the self-circling frequency. (**a**) Depictions of limit cycles at C=0.3, 0.35, and 0.4. (**b**) Self-circling frequency variations with the contraction coefficient.

This section investigates the effect of the contraction coefficient of LCE fibers on the self-circling of the LCE-slider system. The relevant parameter settings are as follows: $\bar{I}=0.3$, $\bar{K}=1.0$, $\bar{v_{0}}=0.9$, $\bar{\beta_{1}}=0.007$, $\bar{\beta_{2}}=0.0006$, $\bar{a}=2.0$, and $\bar{b}=1.9$. When the contraction coefficients are 0.3, 0.35, and 0.4, they all trigger the self-circling of the system, with the corresponding limit cycles shown in Figure 5a. This graphical relationship demonstrates that an increase in the contraction coefficient results in a larger angular velocity of the LCE-slider system’s self-circling. Additionally, Figure 5b depicts the curve illustrating the influence of the contraction coefficient on frequency. We observe that when the contraction coefficient of the LCE fibers reaches a critical threshold of 0.26, it triggers the slider to achieve sustained periodic rotation. When the contraction coefficient of the LCE fibers is below this critical threshold, the system remains in a static state. Furthermore, the frequency of the system’s self-circling increases with the contraction coefficient. This phenomenon occurs because an increase in the contraction coefficient enhances the efficiency of converting the energy absorbed by the LCE fibers into the mechanical energy of the slider, leading to faster rotation and an increase in the frequency of the slider’s rotation. All of the above findings have confirmed that an increase in the contraction coefficient of LCE fibers can effectively enhance the efficiency of converting light energy into mechanical energy.

### 4.3. Effect of Elastic Coefficient of LCE

**Figure 6:**
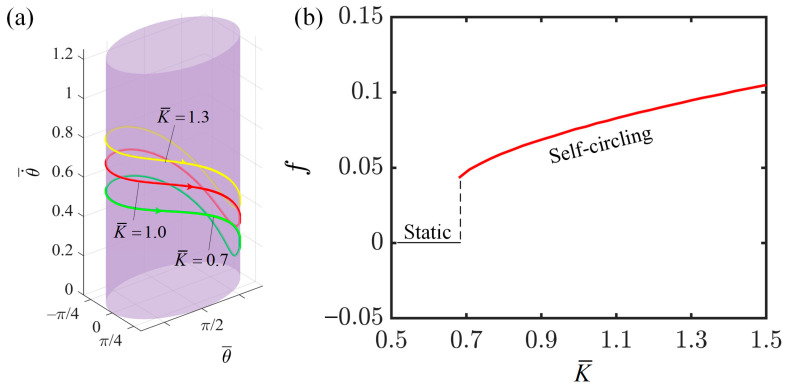
The effect of the elastic coefficient on the self-circling frequency. (**a**) Depictions of limit cycles at $\bar{K}=0.7$, 1.0, and 1.3. (**b**) Self-circling frequency variations with the elastic coefficient.

This section investigates the influence of the elastic coefficient of LCE fiber on the self-circling behavior of the system, with the following relevant parameter settings: $\bar{I}=0.3$, C=0.3, $\bar{v_{0}}=0.9$, $\bar{\beta_{1}}=0.007$, $\bar{\beta_{2}}=0.0006$, $\bar{a}=2.0$, and $\bar{b}=1.9$. The phase trajectory limit cycles in Figure 6a indicate that an increase in the elastic coefficient leads to an increase in the angular velocity of the system’s self-circling. In addition, Figure 6b displays the relationship between the elastic coefficient of the LCE fibers and the frequency of self-circling. When the elastic coefficient reaches 0.68, the net work done by the tension of the LCE fibers on the slider is sufficient to compensate for the system’s energy dissipation, resulting in the system being in a state of self-circling. Conversely, when the elastic coefficient is below 0.68, the net work done by the tension of the LCE fibers on the slider is insufficient to compensate for the system’s energy dissipation, causing the system to remain in a static state. Furthermore, the frequency of self-circling also increases with the elastic coefficient. This is because as the elastic coefficient reaches and exceeds the critical threshold for self-circling; thus, it leads to an increase in the tension of the LCE fibers. Consequently, the driving force acting on the slider to maintain its motion becomes greater, resulting in a higher rotational speed within each cycle of the slider and thus a higher frequency of self-circling. The phase trajectory limit cycles in Figure 6a also confirm that an increase in the elastic coefficient leads to an increase in the angular velocity of the system’s self-circling. This demonstrates that the careful selection and preparation of the LCE with the appropriate elastic coefficient can effectively enhance the energy conversion efficiency of this system.

### 4.4. Effect of Initial Tangential Velocity

**Figure 7:**
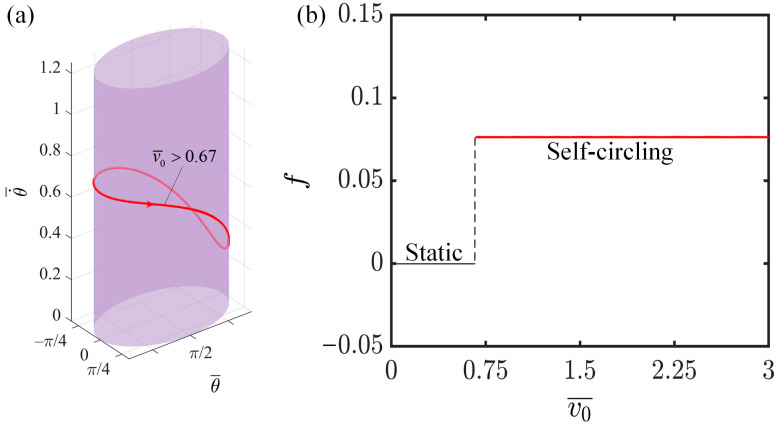
The effect of the initial tangential velocity on the self-circling frequency. (**a**) Depictions of limit cycles at $\bar{v_{0}}=0.9$, 1.2 and 1.5. (**b**) Self-circling frequency variations with initial tangential velocity.

This section primarily investigates the influence of the initial tangential velocity on the self-circling behavior of the system, considering the following relevant parameters: $\bar{I}=0.3$, C=0.3, $\bar{K}=1.0$, $\bar{\beta_{1}}=0.007$, $\bar{\beta_{2}}=0.0006$, $\bar{a}=2.0$, and $\bar{b}=1.9$. When the initial velocity exceeds 0.67, the limit cycles synchronize at various initial velocity levels, as shown in Figure 7a. From Figure 7b, it can be observed that once the initial velocity surpasses the pivotal limit of 0.67, the system acquires the capability to circle toward the illumination and accumulate adequate energy to perpetuate its self-circling; consequently, the system remains in a state of self-circling. In contrast, if the initial velocity does not exceed the critical threshold, the system is unable to access the illumination and gather sufficient energy to balance out the dissipation, resulting in the system remaining in a static state. Furthermore, an increase in the initial velocity does not lead to an increase in the frequency of self-circling. The reason for this is that the frequency of self-circling is dictated by the efficiency at which light energy is converted into mechanical energy, a process that remains unaffected by variations in the initial velocity. Here, the initial velocity serves as a prerequisite for triggering self-circling, activating the system but not controlling its operation. The observation that the initial velocity has no bearing on the system’s self-circling performance indicates the commendable robustness exhibited by the self-circling system.

### 4.5. Effect of the First Damping Coefficient

**Figure 8:**
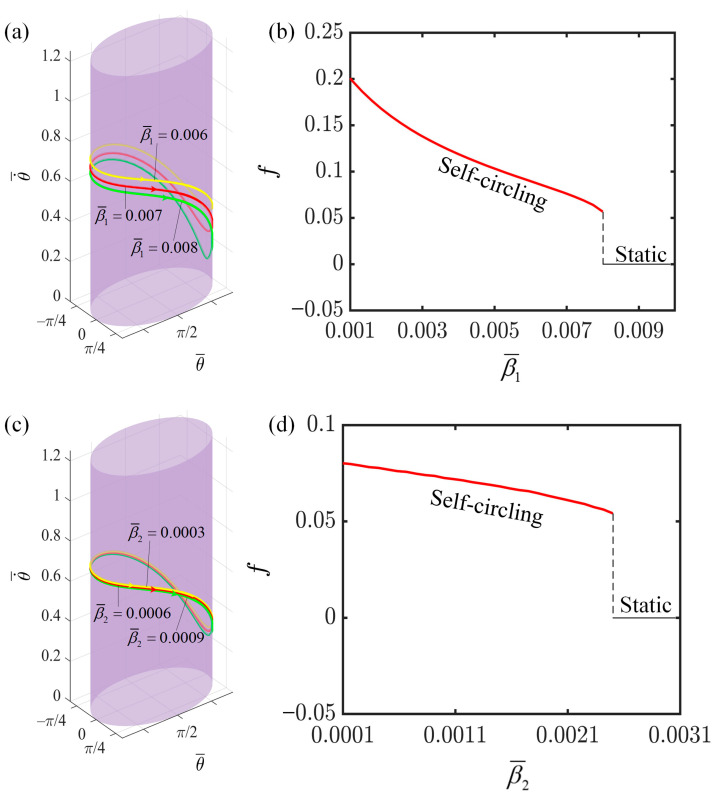
The effect of damping coefficients on the self-circling frequency. (**a**) Depictions of limit cycles at $\bar{\beta_{1}}=0.006$, 0.007, and 0.008. (**b**) Self-circling frequency variations with the first-order damping coefficient $\beta_{1}$. (**c**) Depictions of limit cycles at $\bar{\beta_{2}}=0.0003$, 0.0006, and 0.0009. (**d**) Self-circling frequency variations with the second-order damping coefficient $\beta_{2}$.

This section investigates the influence of damping coefficients $\beta_{1}$ and $\beta_{2}$ on the rotation of the LCE-slider system, with the relevant parameter settings outlined as follows: $\bar{I}=0.3$, C=0.3, $\bar{K}=1.0$, $\bar{v_{0}}=0.9$, $\bar{a}=2.0$, and $\bar{b}=1.9$. In Figure 8a,c, it is evident that the limit cycle for $\beta_{1}=0.006$ is positioned higher than those for $\beta_{1}=0.007$ and $\beta_{1}=0.008$; similarly, the limit cycle for $\beta_{2}=0.0003$ is higher than those for the presumably intended $\beta_{2}$ values of 0.0006 and 0.0009. This underscores that an increase in both the first-order and second-order damping coefficients leads to a reduction in the angular velocity of rotation. Figure 8b specifically examines the effect of $\beta_{1}$ on the rotation frequency of the system. When $\beta_{1}$ is less than 0.0082, the energy absorbed by the system is sufficient to sustain its rotation, keeping the system in a rotating state. However, as $\beta_{1}$ exceeds 0.0082, the increased damping force results in greater energy consumption during the slider’s motion, to the point where the energy absorbed by the LCE fibers from the illuminated region becomes insufficient to maintain the motion, ultimately causing the slider to cease rotating. An analogous explanation holds true for the effect of $\beta_{2}$ on the rotation frequency, as depicted in Figure 8d. Here, it is evident that the critical value for the second-order damping coefficient $\beta_{2}$ is 0.002. If $\beta_{2}$ is less than this threshold, the system remains in a rotating state; otherwise, it becomes static. Moreover, as $\beta_{1}$ or $\beta_{2}$ increase, the force impeding the system’s motion also intensifies, necessitating more energy from the system to counteract this resistance. Consequently, less kinetic energy is available for the slider, leading to a decrease in both the angular velocity and the rotation frequency. Additionally, through comparisons, it becomes apparent that the first-order damping coefficient exerts a more pronounced influence on angular velocity and motion frequency compared to the second-order damping coefficient. This is caused by the resistance Formula (8). Thus, damping coefficients are crucial factors that cannot be overlooked in achieving optimal system performance.

### 4.6. Effect of Semi-Major Axis

**Figure 9:**
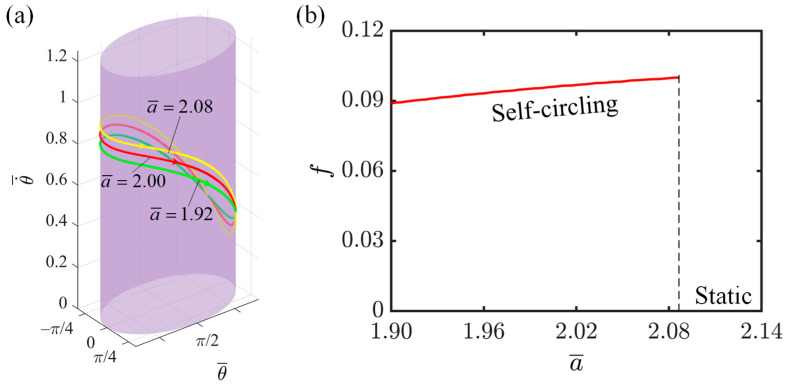
The effect of the elliptical semi-major axis on the self-circling frequency. (**a**) Depictions of limit cycles at $\bar{a}=1.92$, 2.00, and 2.08. (**b**) Self-circling frequency variations with elliptical semi-major axis.

This section investigates the impact of elliptical semi-major axis on the rotation of the LCE-slider system, with relevant parameter settings as follows: $\bar{I}=0.3$, C=0.3, $\bar{K}=1.0$, $\bar{v_{0}}=0.9$, $\bar{\beta_{1}}=0.007$, $\bar{\beta_{2}}=0.0006$, and $\bar{b}=1.9$. The variation in the motion trajectory is primarily achieved by altering the elliptical semi-major axis. As shown in Figure 9a, the limit ring with a semi-major axis of 2.08 is higher than those with semi-major axes of 2.00 and 1.92. This is because, with the length of the minor axis remaining constant, the longer the semi-major axis is, the more pronounced the flattening of the elliptical orbit becomes. In order to maintain a constant area swept per unit time, when the slider approaches the minor axis, the rotation speed of the slider accelerates, resulting in an increase in the overall speed. Figure 9b depicts the influence of the semi-major axis of the ellipse on the rotation frequency. As the semi-major axis of the ellipse increases, the motion frequency of the slider also increases. This is because the flattening of the ellipse requires the slider to traverse a larger length of the orbit at a higher speed within a shorter period of time, increasing the average angular velocity across the entire orbit. Consequently, the time required for the entire rotation cycle decreases, leading to an increase in the rotation frequency. This phenomenon demonstrates the enhanced adaptability and flexibility of the LCE slider system.

### 4.7. Effect of Semi-Minor Axis

**Figure 10:**
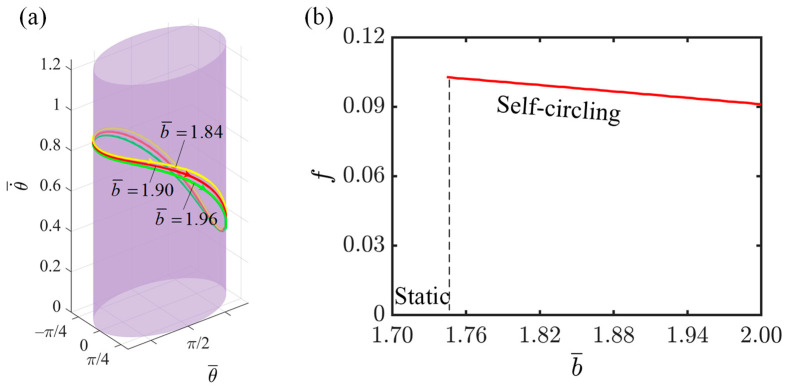
The effect of the elliptical semi-minor axis on the self-circling frequency. (**a**) Depictions of limit cycles at $\bar{b}=1.84$, 1.90, and 1.96. (**b**) Self-circling frequency variations with elliptical semi-minor axis.

This section investigates the impact of elliptical semi-minor axis on the rotation of the LCE-slider system, with relevant parameter settings as follows: $\bar{I}=0.3$, C=0.3, $\bar{K}=1.0$, $\bar{v_{0}}=0.9$, $\bar{\beta_{1}}=0.007$, $\bar{\beta_{2}}=0.0006$, and $\bar{a}=2.0$. The length of the semi-minor axis also affects the degree of flatness of the ellipse. As can be observed from Figure 10a, the limit ring with a semi-minor axis of 1.84 is higher than those with semi-minor axes of 1.90 and 1.96. This is because as the semi-minor axis becomes larger, the flattening of the elliptical orbit becomes less pronounced. Consequently, the speed of the slider slows down as it rotates through the vicinity of the minor axis, leading to a slight decrease in the overall speed. Figure 10b illustrates the impact of the semi-minor axis of the ellipse on the rotation frequency. As the semi-minor axis of the ellipse increases, the motion frequency of the slider gradually decreases. The gradual decrease in the flatness of the ellipse results in the slider traversing the orbit at a slower speed over a short period, reducing the overall average angular velocity of the slider, and subsequently leading to a decrease in the rotation frequency of the slider. By examining the influence of the semi-major and semi-minor axes on the slider’s motion frequency, we discover that as the ratio of the semi-major to semi-minor axes increases, the rotation frequency of the slider also increases. By adjusting the ratio of the semi-major and semi-minor axes of the elliptical orbit, we can optimize the motion characteristics of the system to better suit specific application requirements, providing broader opportunities for the system’s application in various fields.

## 5. Conclusions

Although self-oscillating LCE-slider systems have attracted immense interest owing to their controllability, efficiency, and sustainability and have been widely constructed and developed, a persistent challenge lies in the restriction of system movements solely to circular trajectory, which significantly restricts their full potential. To overcome this limitation and more accurately simulate intricate natural movements, we propose an innovative light-powered LCE-slider system that enables continuous self-circling on an elliptical track and is comprised of a light-powered LCE string, slider, and rigid elliptical track. Based on Newton’s second law and the nonlinear dynamics model of light-powered LCE fiber, coupled with the angle formula in the structure, we have established the dimensionless, dynamic control equations that govern the periodic self-circling behavior of the system. Employing the well-established fourth-order Runge–Kutta method in conjunction with MATLAB R2021a software, we numerically solve the dynamic control equations. Our analysis reveals two distinct behavioral regimes within the LCE-slider system: a static state and a self-circling state. Notably, we provide a detailed examination of the self-circling dynamics and its underlying energy balance mechanism, where the continuous influx of external energy counteracts the energy dissipation due to system damping, thereby preserving the dynamic stability of the system.

In addition, a comprehensive quantitative analysis was conducted, encompassing factors such as light intensity, contraction coefficient, elastic coefficient, initial tangential velocity, damping coefficients, and the elliptical axis ratio. The results of numerical simulations reveal that as the light intensity, contraction coefficient, elastic coefficient, and elliptical axis ratio increase, the self-circling frequency of the LCE-slider system correspondingly rises. However, an increase in the damping coefficient has the opposite effect, causing a substantial reduction in the self-circling frequency. Importantly, when all other factors are kept constant, the velocity and frequency of elliptical motion surpass those of circular motion, indicating more efficient performance. It is also worth mentioning that the initial tangential velocity does not alter the self-circling frequency of the LCE-slider system.

Despite the promising potential for the widespread adoption of the proposed LCE-slider system given its simplicity, efficiency, and adaptability, it is crucial to acknowledge the limitations posed by the small deformation assumptions, the neglect of viscoelastic effects in LCE fibers, and the assumption of illumination regions. To harness the full capabilities of this system for broader applications, future research endeavors will emphasize the integration of viscoelasticity within the model framework, consider variable illumination zones, and explore the dynamic performance of the slider as it traverses various curved tracks in space.

## Figures and Tables

**Table 1:** Material properties and geometric parameters.

Parameter	Definition	Value	Unit
I	Light intensity	0–80	kW/m^2^
C	Contraction coefficient of LCE fiber	0–0.4	/
K	Elastic coefficient of LCE fiber	20–40	N/m
$T_{0}$	*Cis* to *trans* thermal relaxation time	0.02–0.45	s
$\eta_{0}$	Light absorption constant	0.002	m^2^/(s W)
m	Mass of the slider	0–0.02	kg
$v_{0}$	Initial tangential velocity	0–5	m/s
$\beta_{1}$	The first damping coefficient	0–0.3	kg/s
$\beta_{2}$	The second damping coefficient	0–0.15	kg/s^2^
$\theta_{0}$	Range of the illuminated zone	[0, 2π]	rad
a	Semi-major axis	0.01–5	m
b	Semi-minor axis	0.01–5	m
$L_{0}$	Original length of LCE fiber	0.01–5	m

**Table 2:** Dimensionless parameters.

Parameter	$\bar{I}$	C	$\bar{K}$	$\bar{v_{0}}$	$\theta_{0}$	$\bar{\beta_{1}}$	$\bar{\beta_{2}}$	$\bar{a}$	$\bar{b}$
Value	0–1	0–0.4	0–10	0–3	[π, 2π]	0–0.2	0–0.1	0.01–3	0.01–2.9

## Data Availability

The original contributions presented in the study are included in the article/supplementary material, further inquiries can be directed to the corresponding author/s.

## Supplementary Materials

The following supporting information can be downloaded at: https://www.mdpi.com/article/10.3390/polym16162375/s1, Video S1: The process of self-circling of the system.
